# Spinal muscular atrophy as a blueprint for precision therapy in neuromuscular disease

**DOI:** 10.1017/erm.2026.10072

**Published:** 2026-09-23

**Authors:** Busra Cetin, Melike Aliciaslan, Ezgi Erbasan, Busra Cesur Ergun, Salih Sanlioglu

**Affiliations:** Department of Gene and Cell Therapy, Akdeniz University Faculty of Medicine, Türkiye

**Keywords:** gene therapy, Itvisma, nusinersen, onasemnogene abeparvovec, risdiplam, spinal muscular atrophy, zolgensma

## Abstract

**Background:**

Spinal Muscular Atrophy (SMA) is caused by a deficiency of the survival motor neuron (SMN) protein due to loss of *SMN1* and inefficient compensation by *SMN2.* This genetic architecture has driven the development of precision therapeutics. Over the past decade, SMA management has progressed from supportive care to RNA-based splicing modulation and gene replacement therapy.

**Methods:**

This review analyzes the evolution of three therapeutic waves in SMA management: antisense oligonucleotides (nusinersen) for *SMN2* splicing correction; systemic small-molecule splicing modifiers (risdiplam) addressing SMA as a multisystem disorder; and gene replacement therapy (onasemnogene abeparvovec), including recent intrathecal formulations expanding patient eligibility.

**Results:**

Precision therapies have profoundly altered the disease trajectory, especially when administered presymptomatically. Nusinersen sustains splicing correction, risdiplam restores SMN across central and peripheral tissues, and gene replacement offers durable, one-time genetic correction. However, critical challenges persist regarding therapeutic durability, optimal treatment sequencing, systemic involvement, and long-term safety.

**Conclusions:**

The evolution of SMA therapies has transformed neurogenetics. Clinical benchmarks have successfully shifted from reactive, symptomatic management to proactive, molecularly targeted precision medicine.

## Key points

Spinal Muscular Atrophy (SMA) is a genetic disorder caused by a deficiency in the survival motor neuron (SMN) protein due to *SMN1* gene dysfunction; however, its paralog *SMN2* has provided a unique target for precision medicine.

Over the past decade, treatment has rapidly evolved from basic supportive care to advanced therapies including RNA splicing modulators, small molecules, and gene replacement therapies that profoundly alter the disease trajectory.

As the field transitions from acute, life-saving interventions toward lifelong optimization, future management must overcome delivery and safety challenges to achieve proactive, molecular-targeted precision care.

## Introduction: Precision therapeutic strategies and the SMA therapeutic revolution

Spinal muscular atrophy (SMA) exemplifies the success of translational medicine, tracing a path from the initial discovery of the *SMN1* gene to the deployment of disease-modifying molecular therapies even as substantial regional barriers continue to restrict global access. The molecular basis of SMA is uniquely tied to a chromosomal duplication event exclusive to the human lineage (Table 1). Humans possess two nearly identical genes at chromosome 5q13.2, *SMN1* and *SMN2*, positioned telomerically and centromerically, respectively (Ref. 1). While both genes encode the same 294-amino-acid SMN protein, their functional output is vastly different due to a single nucleotide variation (Refs 2, 3). Genetically, approximately 95% of SMA cases are caused by a homozygous deletion of exon 7 in the *SMN1* gene on chromosome 5q, whereas the remaining 5% are caused by compound heterozygosity, with a deletion on one allele and a subtle intragenic mutation on the homologous allele (Refs 4, 5). In the absence of *SMN1*, the body relies entirely on *SMN2* to produce SMN protein. However, *SMN2* is an inefficient backup. A translationally silent C to T transition at position 6 of exon 7 (c.840C>T) in *SMN2* fundamentally alters the splicing process (Figure 1). In *SMN1*, the cytosine at this position facilitates an exonic splicing enhancer (ESE) that attracts the Serine/Arginine-Rich Splicing Factor 1 (SRSF1), ensuring that exon 7 is included in the final mRNA transcript (Ref. 6). In *SMN2*, the thymine substitution disrupts this ESE and simultaneously creates an exonic splicing silencer (ESS) that recruits the inhibitory heterogeneous nuclear ribonucleoproteins (hnRNP*)* A1 and A2 (Ref. 7). Consequently, roughly 90% of *SMN2* transcripts lack exon 7 (*SMN∆7*), producing a truncated protein that is highly unstable and rapidly degraded by the cellular machinery (Ref. 8). Only about 10% of *SMN2* transcripts result in the full-length, functional protein. This marginal production of protein is sufficient for embryonic survival, as a complete absence of SMN is embryonically lethal but insufficient to maintain the health of alpha motor neurons throughout life (Ref. 9). At its core, SMA is caused by a deficiency of SMN protein, a ubiquitously expressed factor essential for the assembly of small nuclear ribonucleoproteins (snRNPs), spliceosomal fidelity, axonal mRNA trafficking, cytoskeletal dynamics, and development and maintenance of the neuromuscular junction (Ref. 10). While the consequences of SMN deficiency are most evident in lower motor neurons, the protein’s systemic expression underlies the expanding recognition of SMA as a multisystem disorder (Refs 11, 12).

**Table 1.** Molecular pathology and therapeutic landscapes of spinal muscular atrophy Table 1. long description.

Genetic determinants of SMA pathogenesis and their functional consequences.				
Genetic factor	Role in SMA pathogenesis	Functional outcome		
*SMN1* Gene	Primary protein producer	High levels of full-length SMN protein		
*SMN2* Gene	Secondary backup gene	Predominantly truncated, unstable protein (SMNΔ7)		
c.840 *C*>*T*	Splicing modifier in SMN2	Disruption of ESE; recruitment of hnRNPA1/A2 silencer		
*ISS* − *N*1	Intronic splicing silencer	Downstream of exon 7; major target for ASO therapy		
*SMN2* Copy number	Phenotypic modifier	Inverse correlation with clinical severity		

Profile of commercialized SMA therapies: From antisense oligonucleotides to gene replacement.				
Therapy	Generic name	Target	Route	Approval status (U.S.)
Spinraza	Nusinersen	*SMN2* splicing (*ISS*–*N1*)	Intrathecal	Dec 2016
Zolgensma	Onasemnogene abeparvovec-xioi	*SMN1* gene replacement	Intravenous	May 2019
Evrysdi	Risdiplam	*SMN2* splicing modifier	Oral	Aug 2020
Itvisma	Onasemnogene abeparvovec-brve	*SMN1* gene replacement	Intrathecal	Nov 2025

**Figure 1.**
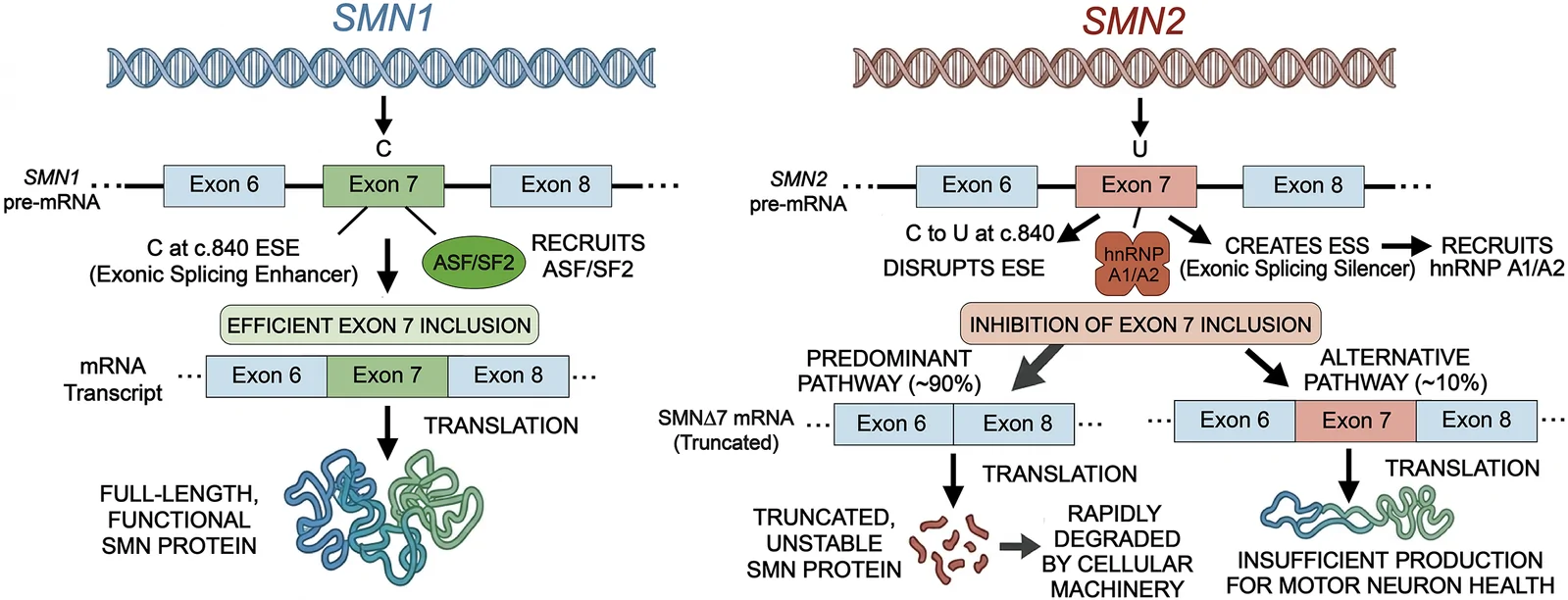
Molecular pathogenesis of SMA via *SMN2* Mis-splicing and systemic protein deficiency. The genetic landscape of SMA is defined by the loss of *SMN1* and the subsequent failure of the *SMN2* backup gene to produce sufficient levels of functional protein. While *SMN1* utilizes a cytosine-mediated exonic splicing enhancer (ESE) to recruit Splicing Factor 2/Alternative Splicing Factor (SF2/ASF) and ensure exon 7 inclusion, a translationally silent c.840C>T transition in *SMN2* disrupts this enhancer while simultaneously creating an exonic splicing silencer (ESS) that recruits inhibitory hnRNP A1/A2 proteins. This molecular switch results in ~90% of *SMN2* transcripts lacking exon 7 (*SMNΔ7*), yielding an unstable, truncated protein that is rapidly degraded, while only ~10% of transcripts produce the full-length, functional protein. Although this marginal production supports embryonic viability, it is insufficient to maintain the health of alpha motor neurons, leading to impaired snRNP assembly, defective axonal mRNA trafficking, and NMJ instability. Given the ubiquitous expression of the SMN protein, this deficiency extends beyond the motor system, characterizing SMA as a multisystem disorder impacting global cellular homeostasis and various organ systems. Figure 1. long description.

Epidemiologically, SMA remains a significant public health concern. It is the leading genetic cause of infant mortality, with an estimated incidence ranging from 1 in 6,000 to 1 in 10,000 live births globally (Ref. 13). In Europe, recent data suggest incidence rates vary between 1 in 3,900 and 1 in 16,000, reflecting geographic and ethnic variability in genetic prevalence (Ref. 14). The carrier frequency of the pathogenic *SMN1* deletion is surprisingly high, estimated between 1 in 40 and 1 in 70 in the general population. This high carrier rate highlights the importance of population-based screening and prenatal counseling.

The management of SMA has transitioned through three distinct therapeutic waves. The first wave focused on symptomatic care and nutritional support. The second wave introduced disease-modifying therapies (DMTs) that target *SMN2* pre-mRNA splicing, whereas the third wave established viral vector-mediated gene transfer to directly replace the functional *SMN1* blueprint (Ref. 15). The standard of care currently includes nusinersen (Spinraza), an antisense oligonucleotide (ASO) administered intrathecally to block an intronic splicing silencer (*ISS-N1*) in *SMN2*, and risdiplam (Evrysdi), an oral small molecule that also promotes *SMN2* exon 7 inclusion. However, the most significant shift toward genetic correction was marked by the global introduction and approval of onasemnogene abeparvovec-xioi (Zolgensma). Depending on the regulatory region, eligibility is defined either by age, such as the US FDA approval for pediatric patients under two years of age, or by clinical characteristics and *SMN2* copy number without a strict age or weight restriction, as seen under European Medicines Agency (EMA) guidelines (Ref. 16). Onasemnogene abeparvovec-xioi delivers a functional *SMN1* transgene via an adeno-associated virus serotype 9 (AAV9) vector, providing rapid onset of SMN protein production (Refs 17, 18).

A critical milestone in this landscape occurred on November 24, 2025, when the FDA approved onasemnogene abeparvovec-brve (Itvisma) for adult and pediatric patients aged 2 years and older (Ref. 19). Onasemnogene abeparvovec-brve utilizes the same AAV9-based vector platform as intravenous onasemnogene abeparvovec-xioi. However, it is administered via the intrathecal (IT) route at a lower total vector dose, enabling targeted delivery to motor neurons while reducing weight-based dosing limitations and systemic toxicities such as hepatotoxicity and cardiotoxicity associated with intravenous administration. As with other AAV9-based therapies, IT delivery does not eliminate the risk of immune responses or overcome limitations associated with redosing. Despite these successes, episomal gene replacement therapies face challenges regarding long-term durability in dividing cells and the lack of endogenous transcriptional control. The transgene remains as an extrachromosomal episome, which may be diluted over time during tissue regeneration or cellular turnover, and its expression is often driven by strong constitutive promoters that do not reflect the cell’s natural physiological requirements.

As of 2026, the therapeutic market is characterized by a high degree of innovation driven by the success of nusinersen, risdiplam, and onasemnogene abeparvovec as outlined in this study (Table 1) (Ref. 20). Here, we provide a comparative analysis of current gene therapies and ASO-based interventions, ultimately outlining critical perspectives on the future of long-term, multidisciplinary patient management.

## The emergence of antisense oligonucleotide therapy

### Nusinersen: Proof of concept for RNA-based therapy

Nusinersen, marketed as Spinraza, represents the first-in-class ASO therapy approved for the treatment of SMA (Ref. 21). Its development was founded on the discovery of the *ISS-N1*, a master regulatory element located downstream of the 5′ splice site of *SMN2* exon 7 (Ref. 22). Targeting this element with a steric-blocking ASO promotes exon 7 inclusion by preventing the binding of inhibitory splicing factors, thereby enhancing full-length SMN protein production without inducing RNase H–mediated degradation of the target RNA (Figure 2). Early preclinical studies using *ISS-N1*–targeting ASOs demonstrated restoration of exon 7 inclusion and increased SMN protein levels in both cellular and animal models of SMA (Refs 23, 24). These interventions resulted in dose-dependent improvements in motor neuron survival and function, establishing a strong proof of concept for RNA-based therapeutic strategies and laying the foundation for the clinical development of nusinersen. Consistent with these preclinical observations, subsequent clinical trials demonstrated significant improvements in motor function and survival in SMA patients treated with nusinersen, supporting its efficacy as a disease-modifying therapy (Refs 25, 26).

**Figure 2.**
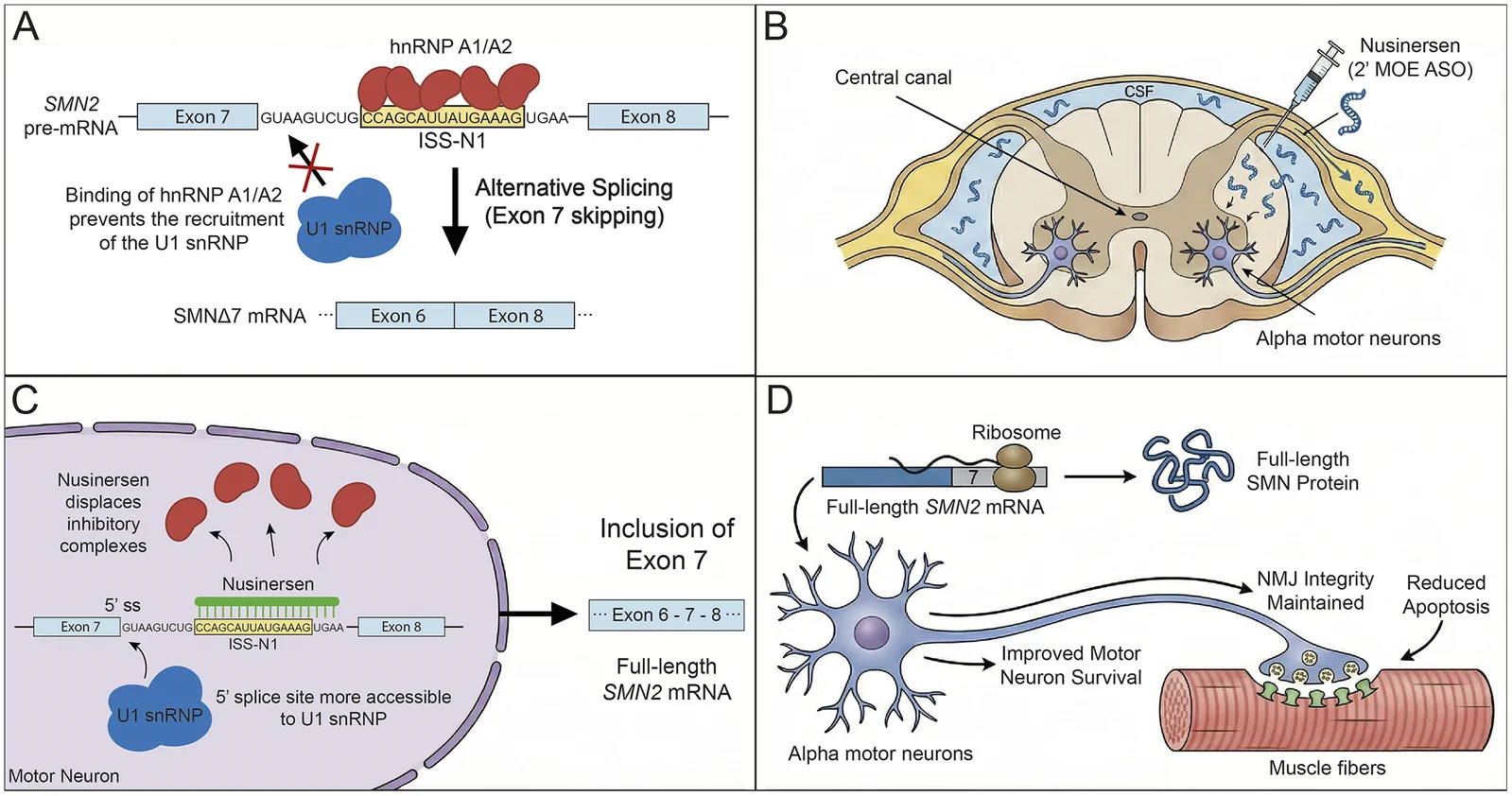
Mechanism of action of nusinersen and its therapeutic effects in SMA. **A**. Schematic representation of *SMN2* pre-mRNA splicing regulation. The intronic splicing silencer N1 (*ISS-N1*), located downstream of exon 7, recruits inhibitory splicing factors such as hnRNP A1/A2, which interfere with the binding of U1 snRNP to the 5′ splice site. This prevents proper spliceosome assembly and results in exon 7 skipping, producing a truncated *SMNΔ7* mRNA transcript. **B.** Intrathecal administration of nusinersen into the cerebrospinal fluid (CSF) enables its distribution throughout the central nervous system, allowing uptake by alpha motor neurons within the spinal cord. **C.** Mechanism of nusinersen action at the molecular level. Nusinersen, a 2′-O-methoxyethyl–modified antisense oligonucleotide, binds specifically to the *ISS-N1* region on *SMN2* pre-mRNA, displacing inhibitory hnRNP complexes. This restores accessibility of the 5′ splice site to U1 snRNP, promoting exon 7 inclusion and generation of full-length *SMN2* mRNA. **D.** Functional consequences of restored SMN protein expression. Translation of full-length *SMN* mRNA leads to increased SMN protein levels in motor neurons, resulting in improved motor neuron survival, maintenance of NMJ integrity, and reduced apoptosis in skeletal muscle. Figure 2. long description.

### Molecular design and resistance to nuclease degradation

The efficacy of nusinersen is inextricably linked to its chemical composition. It is an 18-nucleotide oligonucleotide modified with 2′-O-methoxyethyl (2′-MOE) ribose sugars and a phosphorothioate (PS) backbone to enhance therapeutic utility (Ref. 27). These modifications address the inherent limitations of unmodified oligonucleotides, which are susceptible to rapid degradation by endogenous nucleases and exhibit poor cellular uptake.

The 2’-MOE modification is a widely established chemical modification in ASO platforms. By replacing the 2′-hydroxyl group of the ribofuranosyl ring with a 2-methoxyethyl group, the molecule gains substantial resistance to exonuclease-mediated hydrolysis and improved binding affinity to target RNA (Ref. 28). Furthermore, the 2’-MOE moiety increases the preference for the 3′-endo pucker conformation of the ribose ring, which favors the formation of stable A-form RNA-like double helices. Additionally, the inclusion of a 5-methyl group on cytosine and uracil bases helps to reduce the potential for an innate immune response to the ASO by mimicking endogenous methylation patterns, thereby improving the safety profile (Ref. 29).

The PS backbone modification entails substituting a non-bridging oxygen atom in the phosphate linkage with a sulfur atom. This modification serves a dual purpose, further increasing resistance to nuclease degradation while enhancing binding of the ASO to plasma and cellular proteins. The increased protein binding facilitated by the PS backbone is critical for the drug’s pharmacokinetics, as it enables the molecule to persist within the cerebrospinal fluid (CSF) and spinal cord parenchyma for extended periods.

The pharmacokinetic profile of nusinersen is characterized by high protein binding in the plasma (greater than 94%) compared to the CSF (less than 25%). Following IT administration, it achieves 100% bioavailability. The drug is metabolized via exonuclease hydrolysis and exhibits a prolonged elimination half-life of 135 to 177 days in the CSF and 63 to 87 days in the plasma (Ref. 30).

### Mechanistic modulation of the *ISS-N1* master checkpoint

Following IT administration, nusinersen is distributed through the CSF and taken up by neurons and glial cells via adsorptive endocytosis (Figure 2). Once internalized, it traffics to the nucleus, where it binds to its target sequence on the *SMN2* pre-mRNA. The molecular target, *ISS-N1*, serves as a recruitment site for inhibitory hnRNPs, specifically hnRNP A1 and A2 (Ref. 22).

The binding of hnRNP A1/A2 to *ISS-N1* creates a steric hindrance that prevents the recruitment of the U1 snRNP to the weak 5′ splice site of exon 7 (Ref. 23). By annealing to *ISS-N1*, nusinersen displaces these inhibitory complexes and promotes the inclusion of exon 7 during the spliceosome assembly process. This splicing correction results in the production of full-length SMN protein, which is vital for the biogenesis of snRNPs themselves and the subsequent maintenance of motor neuron function. This mechanism creates a self-reinforcing loop in which restoration of SMN protein levels supports the general splicing machinery of the cell.

### Clinical trajectory and the paradigm shift to high-dose regimens

The NURTURE study (NCT02386553) highlights the current upper threshold of therapeutic benefit achievable with current SMA disease-modifying therapies and provides a compelling proof of principle for pre-symptomatic intervention. Infants treated before the onset of clinical symptoms achieved key motor milestones such as autonomous sitting, standing, and walking within usual developmental timeframes, outcomes that were historically unattainable in untreated SMA (Ref. 31). Outcomes were influenced by *SMN2* copy number, with infants carrying three copies generally achieving more robust motor development than those with two copies. These findings indicate that early correction of SMN deficiency can markedly alter the course of SMA, allowing for near-normal neuromuscular development when treatment is initiated before irreversible motor neuron loss occurs. The initial clinical trials for nusinersen, such as the Phase 3 ENDEAR (NCT02193074) and CHERISH studies (NCT02292537), established its efficacy across a broad range of SMA types, showing significant improvements in motor function and survival (Refs 25, 26).

However, long-term real-world evidence highlighted a notable heterogeneity in patient responses (Refs 32, 33, 34). This variability is commonly attributed to differences in treatment timing and baseline motor neuron reserve, as neurons lost prior to intervention cannot be regenerated, emphasizing the critical importance of early diagnosis through newborn screening.

These observations prompted a critical re-evaluation of the standard 12 mg dosing regimen. Researchers hypothesized that the original dose might represent a conservative minimum rather than a biological optimum (Ref. 35). The Phase 2/3 DEVOTE trial (NCT04089566) was planned to examine the safety and efficacy of an intensified higher-dose regimen (Ref. 36). This regimen consists of an initial intensified dosing period with two 50 mg administrations spaced 14 days apart, transitioning to 28 mg doses given at four-month intervals.

Primary results from Part B of the DEVOTE trial indicated that the high-dose regimen achieved a mean change of +15.1 points on the CHOP-INTEND scale at day 183, compared to a decline of 11.1 points in the sham group, representing a mean difference of 26.19 points. Reported data from high-dose nusinersen studies indicate a 68% reduction in the risk of death or permanent ventilation, along with a marked decrease in plasma neurofilament light chain (NfL) levels by day 183 (Ref. 37). NfL is a biomarker of neuroaxonal injury, and reductions in its levels are interpreted as reflecting decreased neurodegeneration and treatment response (Refs 38, 39). These findings suggest that higher levels of SMN restoration may enhance therapeutic outcomes. In the open-label Part C of the DEVOTE study, patients who transitioned from the 12 mg regimen to the higher dose experienced additional gains in motor function, with a mean increase of 1.8 points on the Hammersmith Functional Motor Scale Expanded (HFMSE) by day 302. While these data demonstrate the biological activity and potential utility of higher ASO exposure, several key caveats must be noted. Part B of the DEVOTE trial primarily evaluated high-dose nusinersen against a matched historical sham control from ENDEAR. Although the study featured a randomized standard-dose 12 mg arm, it lacked the statistical power required to definitively prove the superiority of the high-dose regimen in a direct comparison. Additionally, comparisons with historical cohorts such as CHERISH should be interpreted cautiously because of evolving standards of supportive care and differences in study populations. In patients previously treated with standard-dose nusinersen, transition to the higher-dose regimen has generally been associated with incremental improvements in motor function.

### Next-generation ASOs

#### Salanersen and the optimization of therapeutic persistence

As the SMA therapeutic field progresses, the focus has expanded to address the logistical and procedural burdens associated with lifelong IT therapy. Salanersen (BIIB115) represents a new generation of ASOs designed with advanced chemical modifications that further enhance target affinity and tissue persistence (Ref. 40).

#### Progressing toward once-yearly dosing with salanersen (BIIB115)

The primary objective of salanersen is to enable a once-yearly dosing schedule, a significant improvement over the current four-month interval required for nusinersen. Salanersen incorporates a next-generation 2′-*O*-[2-(methyl-amino)-2-oxoethyl] (N-methyl amide-NMA) ribose modification, which provides superior metabolic stability and approximately three to four times greater potency than nusinersen in human *SMN2* transgenic mouse models. This engineering supports its potential to maintain biological activity over a 12-month period, as evidenced by sustained NfL suppression in interim Phase 1 data (Ref. 41).

#### STELLAR-1 trial: Evaluating durability and clinical impact of pre-symptomatic intervention

The clinical evaluation of salanersen is currently focused on the Phase 3 STELLAR-1 trial (NCT07221669), which investigates its efficacy and safety in treatment-naive, pre-symptomatic infants. The trial enrolls pre-symptomatic infants aged 42 days or younger who possess two or three copies of the *SMN2* gene. The dosing regimen involves 80 mg administered IT at approximately 12-month intervals. For infants with two *SMN2* copies, the primary endpoint is the percentage sitting without support at 12 months, while for those with three copies, it is walking alone at 18 months. Changes in Compound Muscle Action Potential (CMAP) amplitude function as a key biomarker for therapeutic effect, allowing researchers to monitor motor unit function and neuromuscular junction integrity.

### Systemic SMA treatment through small-molecule splicing modification

#### Risdiplam: Advancing oral therapy for SMA

Approved by the FDA in 2020, risdiplam represents a mechanistically and pharmacologically distinct approach to treating SMA (Ref. 42). As an orally bioavailable small molecule, risdiplam achieves systemic distribution, crossing the blood–brain barrier to modulate *SMN2* splicing in both central and peripheral tissues. This systemic activity has contributed to the recognition of SMA as a multisystem disorder rather than one restricted to motor neurons. These features distinguish risdiplam from intrathecal ASOs by enabling continuous, systemic SMN restoration.

#### Structural and molecular basis of bulge repair

At the molecular level, the mechanism of action of risdiplam is best understood in the context of structural stabilization of the *SMN2* exon 7 5′ splice site rather than direct correction of the defining c.840C>T transition. Structural studies have shown that this splice site forms a suboptimal RNA–RNA duplex with U1 snRNA, characterized by a bulged adenine (A − 1) at the exon–intron junction that impairs efficient spliceosome recognition (Figure 3). Risdiplam acts by stabilizing this conformation, effectively repairing the bulge and enhancing U1 snRNP binding, a process referred to as 5′ splice-site bulge repair, thereby promoting exon 7 inclusion (Refs 43, 44). Importantly, the c.840C>T transition does not define the molecular target of risdiplam but instead alters exon 7 regulation, either through disruption of an ESE or creation of a silencer element (Refs 45, 46). In contrast, the *ISS-N1*, located within intron 7, functions as an independent negative regulator. Its blockade improves exon 7 inclusion by shifting the balance of splicing factors toward exon recognition (Ref. 22).

**Figure 3.**
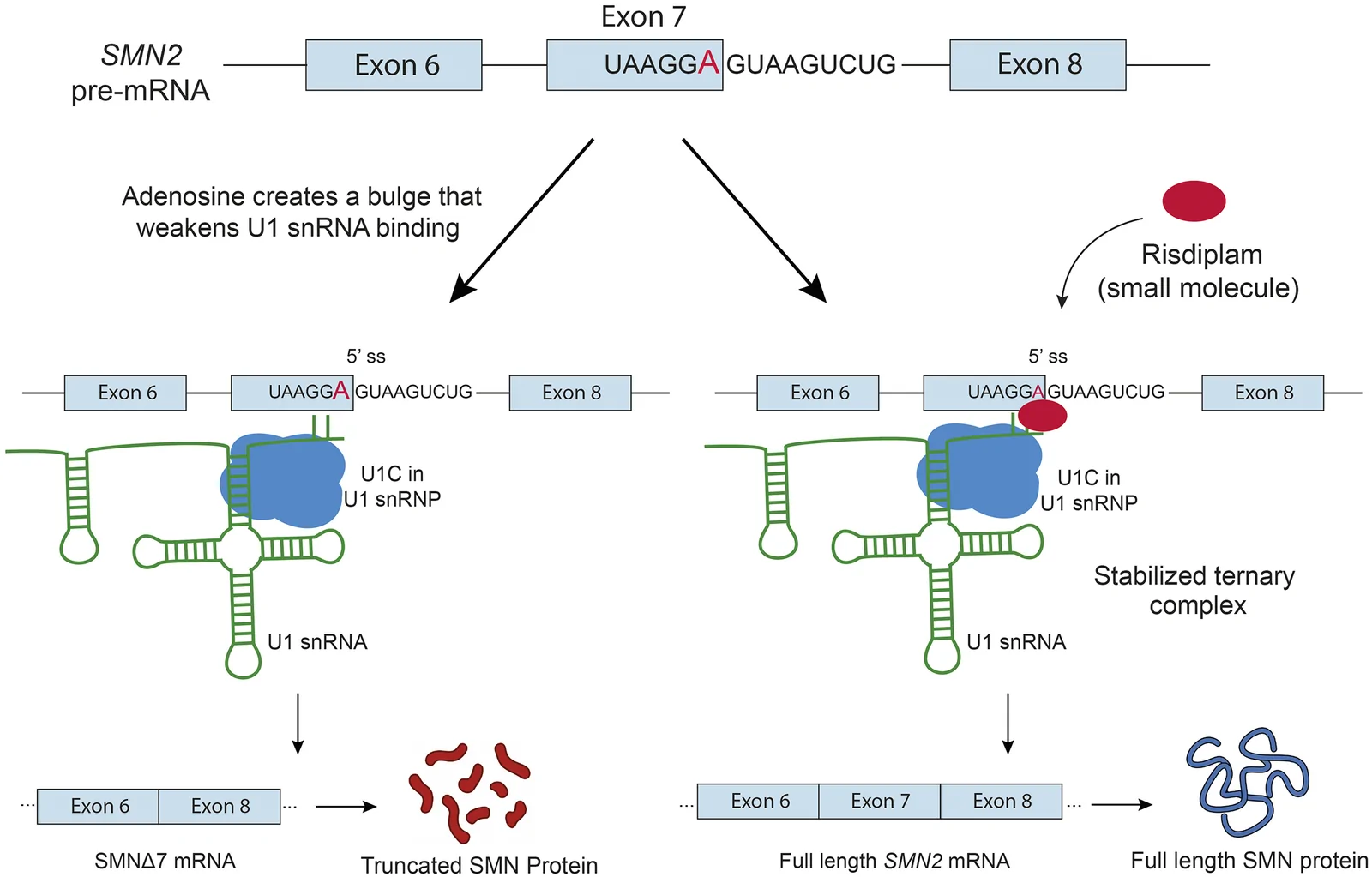
Molecular mechanism of risdiplam–mediated modulation of *SMN2* splicing. Risdiplam is an orally administered, central nervous system penetrant small molecule that modifies *SMN2* pre-mRNA splicing by directly stabilizing interactions within the core spliceosomal machinery. In SMA, exon 7 of *SMN2* is inefficiently included due to an intrinsically weak 5′ splice site at the exon 7–intron 7 junction. This induces a mismatched, bulged RNA structure that sterically hinders U1 snRNP binding, thereby disrupting the earliest stages of spliceosome assembly. Risdiplam binds at the interface between the *SMN2* pre-mRNA 5′ splice site, the U1 snRNP, and the U1-C protein subunit, acting as a molecular bridge that occupies the bulged RNA region. This interaction stabilizes the resulting ternary complex, effectively repairing the mismatched splice site and enhancing U1 snRNP binding. Stabilization of U1 snRNP promotes recruitment of the remaining spliceosome components, functionally converting the weak *SMN2* splice site into a strong, *SMN1*-like splice site. Consequently, exon 7 is retained during mRNA processing, yielding full-length *SMN* mRNA. Translation of this transcript produces stable, functional SMN protein, with increased protein levels achieved in both central nervous system and peripheral tissues, supporting motor neuron survival and systemic cellular homeostasis. Figure 3. long description.

#### Addressing peripheral SMN deficiency in the multisystem pathology of SMA

The systemic distribution of risdiplam is crucial because SMN is a ubiquitously expressed housekeeping protein required for various cellular functions. Peripheral SMN deficiency contributes to broad physiological impairments across multiple systems. In skeletal muscle, it leads to impaired muscle fiber maturation and neuromuscular junction instability. Whether peripheral SMN deficiency directly impairs muscle fiber maturation and neuromuscular junction (NMJ) stability remains a topic of active discussion. While classical genetic studies indicate that NMJ disruption and muscle atrophy are primarily non-cell-autonomous phenomena driven by motor neuron pathology (Refs 47, 48), selective SMN depletion models suggest that intrinsic muscle deficits may further contribute to NMJ instability and compromised fiber maturation (Ref. 49). Metabolic dysregulation is evidenced by abnormalities in glucose, amino acid, and fatty acid metabolism, potentially linked to liver and pancreatic dysfunction. Vascular and cardiac abnormalities have also been described in preclinical models, including reduced microvascular density and developmental defects (Refs 50, 51). Additionally, gastrointestinal and autonomic dysfunction have been documented, and real-world data from adult patients treated with risdiplam have suggested improvements in digestion, appetite, and bulbar function (Ref. 52). Unlike therapies restricted to IT delivery, orally administered risdiplam distributes throughout both central and peripheral tissues, restoring SMN protein levels broadly across multiple organ systems.

#### Long-term efficacy and safety outcomes from the FIREFISH and SUNFISH trials

The long-term durability of risdiplam’s therapeutic effect has been established through five-year data readouts. The five-year readout of the FIREFISH study (NCT02913482) demonstrated that 91% of treated children were alive and 81% survived without permanent ventilation (Refs 53, 54). Functional achievements were significant, with 59% of children sitting unsupported for at least 30 seconds, 21% standing with or without support, and 10% walking with assistance. Furthermore, 96% of treated children retained swallowing ability, and 80% were able to feed independently without a tube. One-year results from the SUNFISH trial (NCT02908685), which included patients aged 2 to 25 years, showed a mean alteration in the 32-item Motor Function Measure (MFM-32) complete score of +1.55 points for the risdiplam group compared to the placebo (Ref. 55). The Revised Upper Limb Module (RULM) score also displayed significant improvement with a 1.59-point difference. Subgroup analysis revealed that 78.1% of children aged 2 to 5 achieved an MFM32 increase of 3 points or more, while 57.1% of patients in the 18 to 25 age group achieved disease stabilization, which is a critical clinical goal in this population. Importantly, no treatment-related adverse events led to study withdrawal in any of the risdiplam trials, and the overall rate of adverse events decreased significantly over the treatment period.

#### The pre-symptomatic frontier and the evolution of sequential therapies

The RAINBOWFISH trial (NCT03779334) emphasizes the transformative potential of early systemic therapy, with infants treated as early as 16 days of age achieving motor milestones comparable to typically developing children (Ref. 56). These findings reinforce newborn screening as an essential component of the SMA care pathway. Furthermore, the JEWELFISH trial (NCT03032172) provided vital insights into the safety of integrating risdiplam into sequential treatment protocols following ASO or gene therapy (Ref. 57). By demonstrating sustained SMN protein elevation and clinical stability, the study reinforces risdiplam’s value as a long-term maintenance or transition choice.

#### Integrating splicing modulation strategies in SMA care

The evolution of nusinersen and risdiplam highlights two fundamentally different but complementary approaches to splicing modulation. Nusinersen utilizes the high-affinity steric blockade of an intronic silencer to achieve targeted rescue within the CNS, while risdiplam employs a systemic small-molecule mechanism to address the multisystemic nature of the disease. The movement toward higher doses and longer-acting formulations, such as the NMA-modified salanersen, reflects a deeper understanding of the biological requirements for SMN protein. As newborn screening becomes more widely implemented, clinical priorities are evolving from managing progression to true disease prevention, pointing toward a future in which SMA no longer ranks among the leading causes of infant mortality.

## Gene replacement therapy

### Onasemnogene abeparvovec-xioi: Pioneering one-time gene therapy for SMA

Gene replacement therapy represents the most direct approach to SMA treatment by introducing a functional *SMN1* transgene to bypass the endogenous genetic defect. Onasemnogene abeparvovec-xioi is the foundational gene therapy agent in this space, utilizing a recombinant AAV9 vector to deliver the human *SMN1* cDNA (formerly AVXS-101) (Ref. 18). The selection of AAV9 was based on its unique capacity to cross the blood–brain barrier and its high tropism for the CNS, particularly motor neurons, when administered systemically or directly into the CSF (Figure 4).

**Figure 4.**
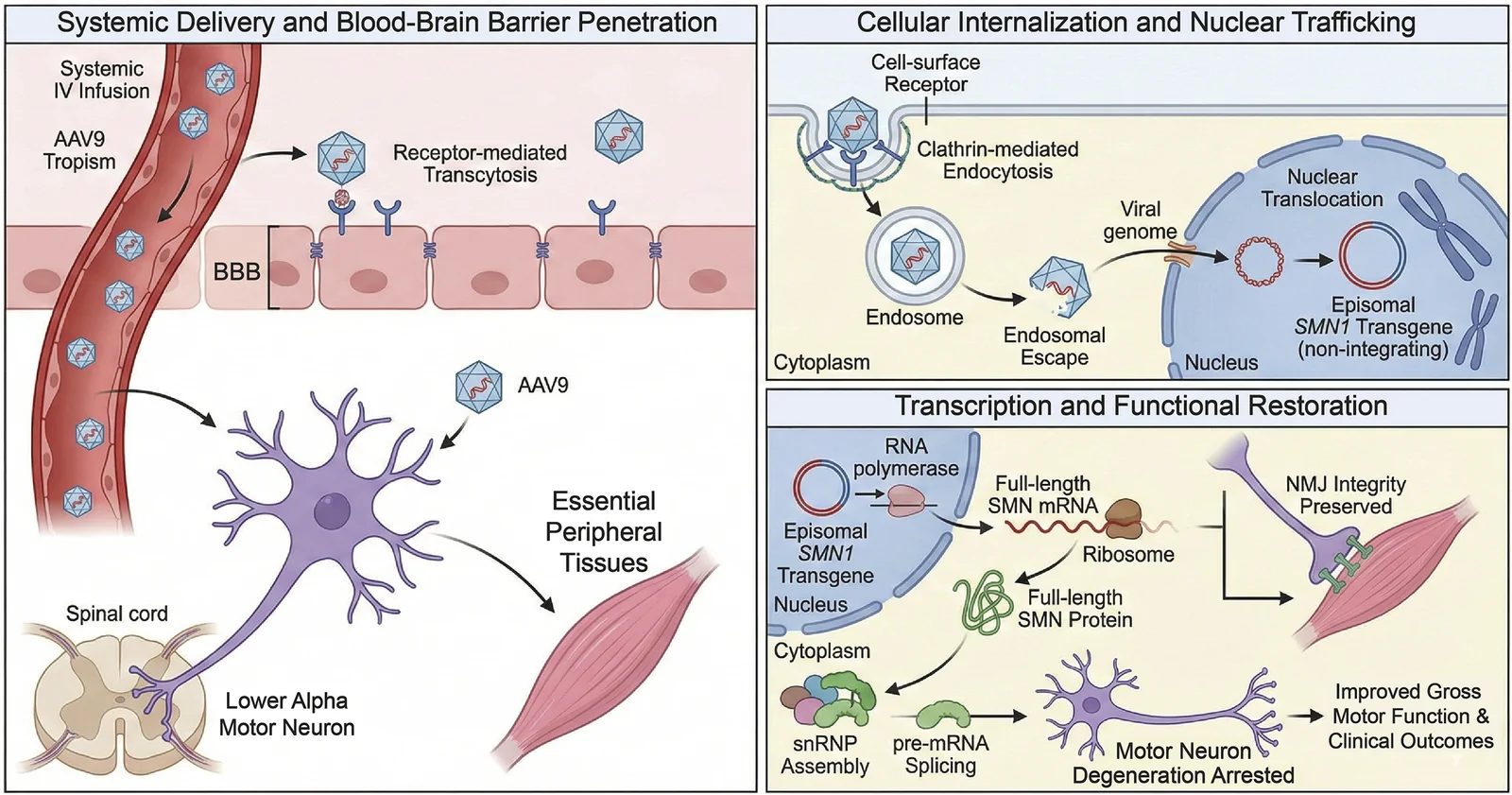
Molecular pathophysiology and mechanism of action of onasemnogene abeparvovec-xioi in SMA. Onasemnogene abeparvovec-xioi (Zolgensma®) is a non-integrating, adeno-associated virus serotype 9 (AAV9)-based gene therapy designed to correct the monogenic deficiency of *SMN1* in SMA. The therapeutic cascade begins with systemic delivery following a single intravenous infusion. Recombinant AAV9 (rAAV9) capsids circulate in the bloodstream and, owing to their intrinsic neurotropism, cross the blood–brain barrier via receptor-mediated transcytosis, enabling transduction of lower alpha motor neurons in the spinal cord as well as peripheral tissues. Following target cell engagement, the vector is internalized through receptor-mediated, clathrin-dependent endocytosis. After endosomal escape, the viral genome is released into the cytoplasm and subsequently translocated into the nucleus. The *SMN1* transgene persists predominantly as a non-integrating episomal DNA molecule, minimizing the risk of insertional mutagenesis while providing a stable template for long-term gene expression, particularly in post-mitotic cells such as motor neurons. Transcription of the episomal *SMN1* transgene by host RNA polymerase results in the production of full-length SMN mRNA, which is translated into functional SMN protein. Restoration of SMN levels supports small nuclear ribonucleoprotein (snRNP) assembly and corrects pre-mRNA splicing defects. At the tissue level, this leads to preservation of NMJ integrity, improved motor neuron survival, and overall enhancement of motor function and clinical outcomes in patients with SMA. Figure 4. long description.

#### Self-complementary genome kinetics

A pivotal advancement in the design of onasemnogene abeparvovec-xioi is the utilization of a self-complementary (scAAV) genome (Ref. 58). Traditional single-stranded AAV vectors require the host cell to synthesize a second, complementary DNA strand before transcription can initiate a process that is often rate-limiting and can delay the onset of therapeutic protein production (Refs 59, 60). In contrast, scAAV vectors package a double-stranded DNA genome by utilizing a mutant inverted terminal repeat that prevents terminal resolution during viral production. This enables the vector to fold back on itself upon entry into the nucleus, forming a transcriptionally active double-stranded template almost immediately. Given the critical therapeutic window in SMA, the fast-acting kinetics of scAAV9 are vital, as the loss of motor neurons is presently irreversible once degeneration occurs. By accelerating the manufacture of SMN protein, onasemnogene abeparvovec-xioi can arrest the neurodegenerative process more effectively than slower-acting genetic interventions.

#### Episomal stability and promoter selection

The *SMN1* transgene delivered by the AAV9 vector persists as an episome within the nucleus of post-mitotic cells. Because motor neurons do not undergo division, these episomes remain stable and capable of sustained protein expression over years, providing the biological basis for the one-time dosing strategy. The transgene is under the control of a constitutive promoter, typically a cytomegalovirus enhancer/chicken β-actin hybrid promoter, which ensures robust and ubiquitous SMN expression throughout the life of the neuron. Although concerns about genomic integration remain, long-term human data indicate that AAV-mediated gene therapy acts predominantly through stable episomal forms, with integration events occurring rarely and randomly and carrying a theoretical, yet unquantified, risk of tumorigenicity (Ref. 61). In addition to episomal persistence, immunological factors further reinforce the one-time dosing paradigm. Initial exposure to the AAV9 capsid elicits a robust humoral immune response, leading to the formation of high-titer neutralizing antibodies (Ref. 62). These antibodies prevent effective re-administration of the vector and may increase the risk of immune-mediated adverse reactions.

#### Long-term efficacy: Insights from a decade of START and LT-001 follow-up

The clinical foundation of onasemnogene abeparvovec-xioi was established through the Phase 1 START trial (NCT02122952), which evaluated intravenous delivery in infants with SMA Type 1 (Ref. 63). This study not only demonstrated safety but also provided the first evidence that gene therapy could fundamentally alter the natural history of the disease. The long-term follow-up study, LT-001 (NCT03421977), has since provided a decade of data on these pioneering patients.

Recent interim data from the LT-001 registry reflecting up to 10 years of post-dose follow-up validated sustained efficacy and safety of onasemnogene abeparvovec-xioi (Ref. 64). All ten patients in the therapeutic dose cohort (1.1 x 10^14^ vg/kg) have survived and remain free from permanent ventilatory support. This is a complete disparity to the ordinary history of untreated SMA Type 1, where 90% of infants typically require permanent ventilation or face mortality by 24 months of age. Crucially, every motor milestone achieved in the initial START study has been sustained throughout the decade-long follow-up. Furthermore, two patients in the therapeutic dose group achieved a new milestone of standing with assistance during the LT-001 period, years after their initial infusion. These findings suggest that early SMN restoration establishes a stable neuromuscular environment capable of supporting continued development as patients age.

In the LT-001 study, 70% of the therapeutic-dose cohort eventually received add-on treatments such as nusinersen or risdiplam. This clinical reality reflects the evolving management of SMA, where gene therapy provides the biological foundation, but clinicians may introduce *SMN2* splicing modifiers to potentially optimize SMN protein levels further, particularly in the periphery or in cases where motor improvements plateau (Ref. 65). Despite the introduction of these adjunct therapies, the underlying stability of the gene therapy effect remains the cornerstone of their long-term survival.

### The regulatory and clinical rationale for IT delivery

While the intravenous formulation of onasemnogene abeparvovec-xioi revolutionized infant care, its application in older, heavier children and adults was constrained. Systemic intravenous dosing is body-weight dependent, and as weight increases, the total viral load required to achieve therapeutic CNS concentrations becomes immense. This high systemic exposure increases the risk of immune-mediated hepatotoxicity and cardiotoxicity, and it can be limited by high pre-existing anti-AAV9 antibody titers.

#### Onasemnogene abeparvovec-brve: Direct CNS targeting

The November 2025 FDA approval of onasemnogene abeparvovec-brve represents the next frontier in gene replacement therapy (Ref. 66). Onasemnogene abeparvovec-brve is an IT formulation specifically indicated for patients 2 years of age and older with *SMN1* mutations. By delivering the AAV9 vector directly into the CSF via a lumbar puncture, onasemnogene abeparvovec-brve bypasses the systemic circulation and achieves concentrated exposure to the spinal cord motor neurons, the primary site of SMA pathology (Figure 5). Direct administration into the CSF allows for a significantly lower total dose of vector than would be required intravenously for an older child or adult. The recommended fixed dose of 1.2 × 10^14^ vector genomes, independent of patient age or body weight, overcomes limitations associated with weight-based dosing and expands treatment eligibility to older and heavier patients. These findings are further supported by clinical evidence demonstrating the safety and feasibility of intrathecal AAV9-mediated gene delivery in SMA populations (Ref. 67).

**Figure 5.**
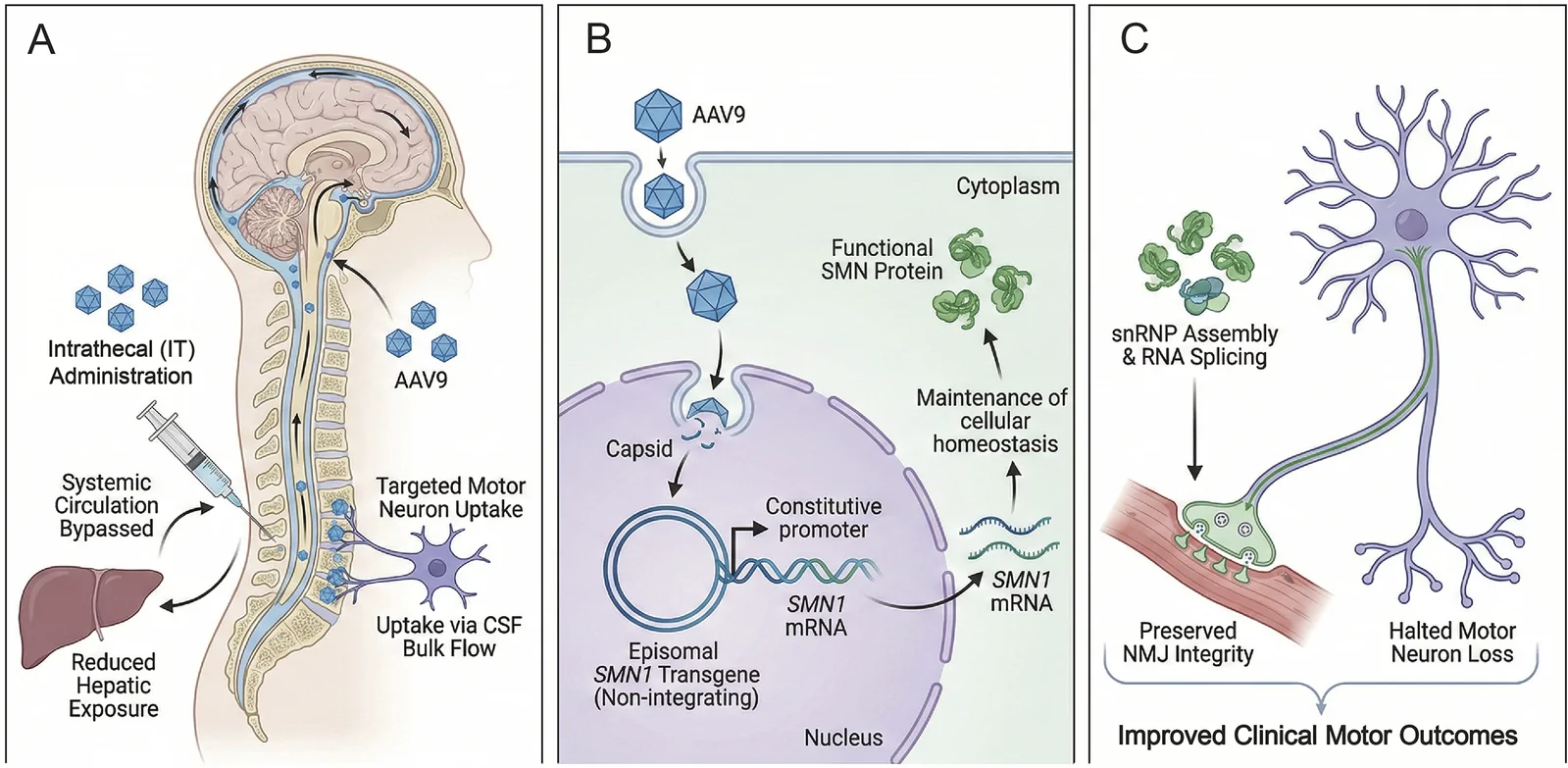
Targeted neuro-axial delivery and mechanism of action of intrathecal onasemnogene abeparvovec-brve (Itvisma) in SMA. **A.** Intrathecal delivery and CNS targeting. Following administration via lumbar puncture, AAV9 vector is introduced directly into the cerebrospinal fluid (CSF). The vector distributes along the neuroaxis via CSF bulk flow, enabling targeted uptake by spinal motor neurons. Compared with systemic intravenous delivery, this route reduces peripheral exposure while facilitating CNS-directed gene transfer, supporting the use of a fixed total vector dose independent of body weight. **B.** Cellular uptake and gene expression. At the cellular level, AAV9 particles are internalized by target cells, followed by capsid uncoating and nuclear delivery of the viral genome. The *SMN1* transgene persists predominantly as a non-integrating episomal DNA molecule. Under the control of a constitutive promoter, this episomal template drives sustained production of full-length SMN mRNA and protein. **C.** Functional restoration and clinical impact. Increased SMN protein levels support small nuclear ribonucleoprotein (snRNP) assembly and restore pre-mRNA splicing homeostasis. At the tissue level, this leads to preservation of neuromuscular junction (NMJ) integrity, stabilization of motor neuron survival, and improvement in motor function and clinical outcomes in patients with SMA. Figure 5. long description.

#### Clinical outcomes from the STEER and STRENGTH pivotal trials of onasemnogene abeparvovec-brve

The efficacy and safety of intrathecal onasemnogene abeparvovec-brve were demonstrated in the phase 3 STEER study (NCT05089656), which evaluated treatment-naive patients, and were supported by the phase 3b STRENGTH study (NCT05386680), which evaluated treatment-experienced patients who transitioned from prior SMN-targeted therapies. These trials aimed to demonstrate that gene therapy could achieve meaningful motor function gains even in later disease stages. STEER (NCT05089656) was a randomized, double-blind, sham-controlled study of 126 patients aged 2 to 17 years who were sitting non-ambulatory at baseline (Ref. 19). Participants received either a single IT dose of onasemnogene abeparvovec-brve or a sham procedure and were monitored for 52 weeks. The primary endpoint was the alteration in the HFMSE scores. This scale is an objective measure of motor ability, where a change of 2 to 3 points is accepted clinically meaningful in older patients who are typically in a state of gradual decline (Ref. 68). The STEER trial proved that onasemnogene abeparvovec-brve could elicit motor gains typically not seen in the natural history of the illness. The stabilization and improvement in HFMSE scores represent a critical shift from preventative to modifying therapy in an older population.

The Phase 3b STRENGTH study (NCT05386680) focused on a real-world cohort of 27 patients aged 2 to 18 years who had previously been treated with and discontinued nusinersen or risdiplam (Ref. 67). This open-label study was the first to test the IT formulation in a treatment-experienced population. At week 52, patients in STRENGTH demonstrated a mean increase of 1.05 points in HFMSE scores. Although the gains were more modest than those observed in the treatment-naive STEER cohort, they nonetheless confirmed motor function stabilization as a successful outcome in this advanced population. Furthermore, three participants achieved new milestones, including standing with assistance and walking independently. These findings support the use of onasemnogene abeparvovec-brve as a viable option for patients who may be dissatisfied with their current chronic therapy or seeking the potential benefits of genomic stabilization.

#### Physiological factors influencing biodistribution and tissue barriers

The delivery of AAV9 via the IT route involves complex physiological interactions. While direct CSF delivery targets the spinal cord, the vector is not entirely sequestered within the CNS. Following lumbar puncture, the AAV9 vector enters the CSF, where it must traverse the pial surface to reach the parenchyma of the spinal cord and brain. Studies in non-human primates (NHPs) have shown that AAV9-based vectors achieve widespread CNS biodistribution following IT delivery, including transduction of the spinal cord and multiple brain regions (Ref. 69). scAAV9 vectors have further been shown to mediate robust transduction of motor neurons across cervical to lumbar segments, as well as supraspinal regions such as the hippocampus and cerebellum. Notably, most capsids rapidly exit the CSF and enter the peripheral circulation. This peripheral redistribution is a double-edged sword. On one hand, it allows for some cross-correction of non-CNS tissues, such as skeletal muscle or the heart, which also require SMN protein for optimal function. On the other hand, it means that even IT administration can result in systemic effects, including liver enzyme elevations and immune responses.

AAV9’s ability to cross the blood–brain barrier is mediated by receptor-mediated transcytosis across the brain microvascular endothelial cells (Ref. 70). When given intravenously, the vast majority of the dose is cleared by the liver, necessitating high titers to ensure a fraction reaches the brain. IT delivery bypasses this initial barrier, theoretically allowing therapy even in the presence of higher systemic anti-AAV9 antibodies. While the current onasemnogene abeparvovec-brve label maintains a titer cutoff of ≤1:50, NHP research indicates that even massive systemic antibody titers do not impact the efficiency of spinal cord transduction via the IT route (Ref. 71). This suggests that as the clinical community gains more experience with onasemnogene abeparvovec-brve, the strict titer requirements might eventually be relaxed for IT delivery, potentially opening the door for patients who were previously disqualified by natural AAV9 immunity.

#### Safety assessment and toxicological risk mitigation

The safety profile of onasemnogene abeparvovec-brve is largely consistent with the established risks of the AAV9 platform, including hepatotoxicity, thrombocytopenia, and cardiotoxicity, as previously reported in clinical studies of systemic AAV9 gene transfer (Ref. 72). Hepatotoxicity remains the most frequent serious adverse event. It is believed to be triggered by an innate immune response to high vector load in hepatocytes, leading to transaminase (ALT/AST) elevations. To mitigate this, a mandatory corticosteroid regimen is required. If liver function remains unremarkable at the end of the 30-day post-injection period, the dose is tapered over the next four weeks. If transaminases exceed 2x the upper limit of normal, the 1 mg/kg dose is maintained until stabilization. In both the STEER and STRENGTH trials, transaminase elevations were transient and manageable with this protocol, with no reported cases of acute liver failure.

A specific concern for IT gene therapy is dorsal root ganglia (DRG) toxicity. The DRGs, which house the cell bodies of sensory neurons, are highly susceptible to AAV9 transduction. In NHP models, IT delivery has been linked to neuronal degeneration, necrosis, and mononuclear cell infiltration in the DRGs (Ref. 73). While NHPs often show no clinical sensory deficits despite histological lesions, human patients must be monitored for symptoms such as numbness, tingling, or loss of sensation. The inclusion of warnings for peripheral sensory neuropathy in the onasemnogene abeparvovec-brve label reflects this emerging understanding of the sensory neuron’s vulnerability to concentrated AAV9 exposure.

In the context of hematologic and cardiac risk, a transient decline in platelet counts commonly occurs during the first week post-injection. Patients also require monitoring for thrombotic microangiopathy, characterized by hemolytic anemia and acute kidney injury. Cardiac toxicity with troponin-I elevation has been reported and, even in the absence of symptoms, should prompt cardiology consultation if levels do not normalize.

#### Integrated therapeutic strategies and the evolving treatment landscape

The introduction of onasemnogene abeparvovec-brve has expanded the clinical decision-making framework for SMA, requiring a more nuanced approach to sequential and combination therapies (Ref. 74). Sequential therapy, in which a patient moves from a chronic DMT such as nusinersen or risdiplam to gene therapy, is increasingly common. Real-world evidence indicates that children who transition to onasemnogene abeparvovec achieve clinically meaningful motor improvements, although these gains are generally smaller than those observed in treatment-naive patients. The rationale for switching often includes treatment fatigue from periodic IT injections or daily oral medication, and the desire for a one-time genetic correction.

Combination therapy involves using *SMN2*-targeting agents after gene therapy. This approach is being formally evaluated in the RESPOND trial (NCT0448133), which assesses nusinersen in patients who had a suboptimal clinical response to onasemnogene abeparvovec-xioi. Interim results show that many of these patients experience additional gains in motor function and stability in bulbar and respiratory function after adding nusinersen. This suggests that, for some patients, combining gene replacement with splicing modulation may be required to achieve optimal clinical outcomes.

The SMA field continues to innovate beyond current therapies. Research is underway on next-generation vectors, such as covalently closed-end double-stranded AAV (cceAAV), which eliminate the mutant Inverted Terminal Repeat (mITR) resolution step and may provide even higher transgene expression levels with lower doses (Ref. 75).

As SMA increasingly resembles a chronic neuromuscular condition, residual impairments have become more apparent. Even optimally treated patients may experience weakness, fatigue, and limited endurance due to developmental deficits established prior to therapy. This has driven interest in therapies that act downstream or independently of SMN. For example, non-SMN therapies like apitegromab (a myostatin inhibitor) are being investigated as adjuncts to improve muscle strength in patients already stabilized on gene therapy or splicing modifiers (Ref. 76). By enhancing muscle mass and strength, it aims to amplify functional outcomes achieved through SMN restoration. Beyond primary disease-modifying interventions, adjunctive approaches aimed at preserving neuromuscular unit integrity are emerging as an important complementary strategy. In this context, agents such as NMD670, which enhance neuromuscular junction stability, are being investigated as potential adjunctive therapies to augment and sustain the benefits of gene-directed treatments (Ref. 77). Although regulatory delays have occurred, its development signals a broader recognition that optimal SMA care will likely require combination therapy addressing both neuronal survival and muscle performance. The field is thus transitioning from life-saving intervention to lifelong functional optimization, mirroring trajectories seen in oncology and metabolic disease management.

The expansion of gene therapy to all age groups via onasemnogene abeparvovec-brve, combined with the maturation of long-term survival data and the exploration of combination regimens, has fundamentally shifted the prognosis for those living with SMA. While it is not yet a cure in the sense of complete restoration of lost neurons, it represents a definitive disease-modifying achievement that offers the potential for independence and improved quality of life throughout the lifetime. The future of SMA care rests on a precision-based strategy that aligns genetic and biological interventions with the patient’s specific disease stage.

While AAV-mediated gene therapy has transformed the therapeutic landscape of SMA, the reality of life-threatening risks and severe adverse events (SAEs) must be acknowledged (Refs 78, 79). High systemic vector doses trigger significant host immune responses that can precipitate severe organ toxicities (Refs 80, 81). Foremost among these is severe acute liver injury and acute liver failure (Ref. 82); post-marketing case reports have documented fatal liver failure in pediatric patients following corticosteroid tapering (Ref. 72). Additionally, systemic AAV infusion carries the risk of thrombotic microangiopathy (TMA), a life-threatening, complement-mediated condition characterized by hemolytic anemia, severe thrombocytopenia, and acute kidney injury, which has occasionally resulted in fatal outcomes despite therapeutic intervention (Ref. 83). Other reported SAEs include DRG toxicity, acute cardiac injury, and systemic hyperinflammatory responses (Refs 84, 85). These rare but severe outcomes highlight that gene therapy carries non-negligible, life-threatening risks, necessitating rigorous pre-screening, tailored immunosuppressive prophylaxis (e.g., high-dose systemic corticosteroids), and vigilant post-infusion clinical and laboratory surveillance.

## Discussion and future perspectives: Comparative analysis of gene replacement and RNA-targeted therapeutics in SMA

Gene therapy is described as a therapeutic strategy that involves the transfer of genetic material into patient cells with the intent to replace, supplement, or modify gene function in order to treat or prevent disease (Ref. 86). Central to this definition is the introduction of exogenous nucleic acid, typically DNA, that enables sustained expression of a functional gene product and directly addresses the underlying genetic defect. Gene therapies are therefore distinguished by their ability to alter gene function at its source and by the potential for long-lasting biological effects following a single or limited number of administrations (Ref. 87). Onasemnogene abeparvovec (both intravenous -xioi and intrathecal -brve formulations) qualifies as a true gene replacement therapy because it uses an AAV9 vector to deliver functional *SMN1* DNA into target cells. Persisting episomally, this exogenous DNA drives sustained, long-term SMN protein production after a single dose by directly addressing the root genetic cause of SMA. In contrast, nusinersen and risdiplam are non-gene-therapy disease-modifying agents that act strictly at the RNA/pharmacologic level without altering or introducing genetic material. Nusinersen (an intrathecal ASO) and risdiplam (an oral small molecule) temporarily modify *SMN2* pre-mRNA splicing to promote exon 7 inclusion, requiring repeated, lifelong administration to sustain protein expression. These fundamental distinctions directly influence therapeutic durability, administration regimens, longitudinal safety monitoring, and the broader clinical and ethical implications of lifelong medical care.

Despite transformative clinical breakthroughs, the long-term management of SMA faces several unresolved challenges. Structurally, it remains undetermined what precise degree, biodistribution, and duration of SMN restoration are necessary to maintain lifelong neuromuscular stability, and whether these therapeutic thresholds diverge across developmental stages. Similarly, the biological consequences of chronic, supraphysiologic SMN expression especially within presymptomatically treated cohorts remain uncharacterized. Clear clinical guidelines are also lacking regarding the optimal sequencing, combination, or switching of SMN-directed platforms, leaving the long-term implications of incomplete or waning therapeutic efficacy over decades undefined. Furthermore, the degree to which embryonic or early postnatal SMN deficiency irreversibly imprints upon motor circuit architecture, neuromuscular junction maturation, and intrinsic muscle biology remains an open question. This clinical ambiguity is compounded by a deficit in validated biomarkers capable of tailoring treatment intensity and predicting longitudinal functional outcomes. Finally, as the treated population enters adulthood, the long-term natural history of altered SMA remains a critical blind spot, particularly regarding age-related vulnerabilities, cumulative treatment burdens, and the lifetime durability of current interventions.

In conclusion, the next decade of SMA management will transition from reactive, life-saving interventions to proactive, lifelong optimization. As the field matures beyond basic SMN restoration, clinical focus is shifting toward the refinement of treatment timing, delivery vectors, and biomarker-guided monitoring. Early identification and timely treatment will remain central to achieving optimal outcomes (Ref. 88). While novel gene-editing platforms provide a conceptual pathway for permanent correction (Refs 89, 90, 91, 92, 93), substantial delivery and safety constraints persist. Addressing these challenges necessitates adaptive therapeutic algorithms that evolve alongside the patient’s developmental stage and long-term safety profile. Moving forward, clinical endpoints must place greater weight on functional durability and systemic participation across the lifespan rather than transient milestone achievements alone. Through this transition, the evolution of SMA care will redefine neurogenetics, cementing its role as a model for precision medicine in chronic genetic disorders.

## Data Availability

Data sharing does not apply to this article, as no new datasets were generated or analyzed during the current study.

## Literature search strategy and selection criteria

A comprehensive literature search was conducted across the PubMed, Embase, and MEDLINE databases to identify peer-reviewed articles published from database inception through July 2026. The search strategy utilized combinations of Medical Subject Headings (MeSH) terms and high-impact keywords, including: spinal muscular atrophy, SMA, nusinersen, onasemnogene abeparvovec, Itvisma, risdiplam. Articles were screened sequentially by title, abstract, and full text, and selected for inclusion based on their direct relevance to the molecular pathology of SMA, clinical trial outcomes, and pharmacokinetic/pharmacodynamic profiles. Priority was given to randomized controlled trials (RCTs), prospective cohort studies, long-term registry updates (e.g., LT-001), and definitive preclinical non-human primate (NHP) or murine modeling data.

## Long descriptions

### Table 1. Long description

Panel A. Genetic determinants of SMA pathogenesis and their functional consequences. Panel A presents the major genetic determinants involved in SMA pathogenesis and their functional consequences. The table includes three columns: Genetic Factor, Role in SMA Pathogenesis, and Functional Outcome. The first row describes the SMN1 gene as the primary producer of survival motor neuron (SMN) protein, resulting in high levels of full-length functional SMN protein. The SMN2 gene is identified as a secondary backup gene that predominantly generates a truncated and unstable SMNΔ7 protein due to alternative splicing. The c.840C>T synonymous transition in SMN2 is described as a critical splicing modifier that disrupts an exonic splicing enhancer (ESE) and promotes recruitment of the hnRNPA1/A2 silencer complex, leading to exon 7 exclusion. The intronic splicing silencer ISS-N1 is highlighted as a major regulatory element downstream of exon 7 and an important therapeutic target for antisense oligonucleotide-based therapies. Finally, SMN2 copy number is presented as an important phenotypic modifier, showing an inverse correlation with clinical severity, where higher copy numbers are generally associated with milder disease phenotypes.

Panel B. Profile of commercialized SMA therapies: From antisense oligonucleotides to gene replacement. Panel B summarizes commercially approved SMA therapies and compares their mechanisms and regulatory status. The panel contains the columns Therapy, Generic Name, Target, Route, and Approval Status (U.S.). The first therapy, Spinraza (nusinersen), targets SMN2 pre-mRNA splicing through antisense oligonucleotide technology and is administered intrathecally; it is FDA approved. The second therapy, Zolgensma (onasemnogene abeparvovec), is a gene replacement therapy delivering a functional SMN1 transgene using an AAV9 vector via intravenous administration and is also FDA approved. The third therapy, Evrysdi (risdiplam), is a small-molecule splicing modifier that enhances exon 7 inclusion in SMN2 transcripts and is administered orally; it is likewise FDA approved. Itvisma (onasemnogene abeparvovec-brve): A localized gene replacement therapy that uses the same functional SMN1 transgene and AAV9 vector as Zolgensma, but is formulated specifically for a single intrathecal (spinal) injection. It is FDA approved for adults, teens, and children aged two years or older, providing a weight-independent gene therapy option for older patient populations.

Navigate back to Table 1..

### Figure 1. Long description

The diagram is divided into two main vertical sections.

Left Panel: SMN1

- Top: A blue double-helix DNA strand labeled SMN1.

- Middle: SMN1 pre-mRNA shows Exon 6, Exon 7 (green), and Exon 8. A ‘C’ nucleotide at c.840 ESE (Exonic Splicing Enhancer) recruits ASF/SF2 (green oval).

- Flow: This leads to ‘EFFICIENT EXON 7 INCLUSION’.

- Bottom: The mRNA transcript includes Exon 6, Exon 7, and Exon 8. Translation results in a large, complex blue and green ‘FULL-LENGTH, FUNCTIONAL SMN PROTEIN’.

Right Panel: SMN2

- Top: A red double-helix DNA strand labeled SMN2.

- Middle: SMN2 pre-mRNA shows Exon 6, Exon 7 (red), and Exon 8. A ‘U’ nucleotide at c.840 disrupts the ESE and creates an ESS (Exonic Splicing Silencer), which recruits hnRNP A1 /A2 (brown clover shape).

- Flow: This causes ‘INHIBITION OF EXON 7 INCLUSION’, splitting into two pathways.

- Predominant Pathway (approximately 90 percent): Produces SMN delta 7 mRNA (Truncated) containing only Exon 6 and Exon 8. Translation yields a ‘TRUNCATED, UNSTABLE SMN PROTEIN’ shown as small red fragments that are ‘RAPIDLY DEGRADED BY CELLULAR MACHINERY’.

- Alternative Pathway (approximately 10 percent): Produces a full mRNA transcript (Exons 6, 7, and 8). Translation yields a functional protein, but the diagram notes ‘INSUFFICIENT PRODUCTION FOR MOTOR NEURON HEALTH’.

Navigate back to Figure 1..

### Figure 2. Long description

The diagram is divided into four panels labeled A through D.

* Panel A: A schematic of SMN2 pre-mRNA splicing regulation. At the top, hnRNP A1 /A2 inhibitory factors bind to the I SS-N1 region between Exon 7 and Exon 8. A red cross indicates that this binding prevents U 1 snRNP from reaching the 5 prime splice site. An arrow labeled Alternative Splicing (Exon 7 skipping) points down to the resulting SMN delta 7 mRNA transcript containing only Exon 6 and Exon 8.

* Panel B: An anatomical cross-section of the spinal cord. A syringe labeled Nusinersen (2 prime M O E A S O) injects the drug into the CSF. Blue helical strands represent the drug distributing through the CSF and being taken up by alpha motor neurons located near the central canal.

* Panel C: A molecular view inside a motor neuron. Nusinersen is shown as a green comb-like structure binding to the ISS-N1 region on the SMN2 pre-mRNA. This action displaces the red hnRNP inhibitory complexes. Below, U 1 snRNP successfully binds to the 5 prime splice site. An arrow points to the Inclusion of Exon 7, resulting in full-length SMN2 mRNA containing Exons 6, 7, and 8.

* Panel D: The functional consequences. At the top, a ribosome translates the full-length SMN2 mRNA into full-length SMN protein. This protein supports an alpha motor neuron, leading to improved motor neuron survival. The neuron’s axon extends to muscle fibers where NMJ integrity is maintained and skeletal muscle apoptosis is reduced.

Navigate back to Figure 2..

### Figure 3. Long description

The diagram is organized into three vertical stages.

1. Top Stage: SMN2 pre-mRNA structure.

A horizontal line represents the pre-mRNA containing Exon 6, Exon 7, and Exon 8. Exon 7 contains the sequence UAAGGA followed by GUAAGUCUG. A red letter A in the sequence is highlighted as the adenosine that creates a bulge, weakening U1 snRNA binding.

2. Middle Stage: Comparison of spliceosome assembly.

* Left side (Untreated): The U1 snRNA (green cloverleaf structure) and U1 C protein (blue shape) attempt to bind at the 5 prime ss junction. The red adenosine bulge prevents a stable connection, leading to an arrow pointing down to mRNA lacking exon 7.

* Right side (Risdiplam treatment): A red oval representing Risdiplam is positioned at the interface of the 5 prime ss, the U1 C protein, and the U1 snRN A. This forms a stabilized ternary complex, bridging the gap caused by the red adenosine bulge.

3. Bottom Stage: Final products.

* Left side: SMN delta 7 mRNA (Exon 6 joined directly to Exon 8) leads to fragmented red shapes labeled Truncated SMN Protein.

* Right side: Full length SMN2 mRNA (Exon 6, Exon 7, and Exon 8) leads to a large blue coiled structure labeled Full length SMN protein.

Navigate back to Figure 3..

### Figure 4. Long description

Panel 1: Systemic Delivery and Blood-Brain Barrier Penetration. A blood vessel shows systemic IV infusion of AAV9 capsids. Arrows indicate AAV9 tropism as capsids move from the vessel through the BBB via receptor-mediated transcytosis across endothelial cells. Outside the vessel, AAV9 vectors target a lower alpha motor neuron in the spinal cord and essential peripheral tissues like muscle.

Panel 2: Cellular Internalization and Nuclear Trafficking. At the cell surface, an AAV9 capsid binds to a cell-surface receptor and is internalized via clathrin-mediated endocytosis into an endosome. Following endosomal escape, the viral genome is released into the cytoplasm and undergoes nuclear translocation through a nuclear pore. Inside the nucleus, the genome forms a circular episomal SMN1 transgene that is non-integrating.

Panel 3: Transcription and Functional Restoration. Inside the nucleus, RNA polymerase transcribes the episomal SMN1 transgene into full-length SMN mRNA. The mRNA exits to the cytoplasm where a ribosome translates it into full-length SMN protein. This protein supports snRNP assembly and corrects pre-m RNA splicing. These molecular changes lead to preserved NMJ integrity at the neuromuscular junction, arrested motor neuron degeneration, and improved gross motor function and clinical outcomes.

Navigate back to Figure 4..

### Figure 5. Long description

The diagram is divided into three panels.

Panel A, titled Intrathecal IT Administration, shows a sagittal view of a human head and spine. A syringe performs a lumbar puncture, injecting blue hexagonal AAV9 particles into the spinal canal. Arrows indicate the flow of CSF bulk flow carrying the vector upward toward the brain and downward. To the left, a liver icon with a curved arrow indicates Systemic Circulation Bypassed and Reduced Hepatic Exposure. To the right, AAV9 particles are shown entering a motor neuron, labeled Targeted Motor Neuron Uptake.

Panel B, titled Cellular uptake and gene expression, shows a cross-section of a cell. An AAV9 particle enters the cytoplasm via endocytosis. It moves to the nucleus where the capsid uncoats. Inside the nucleus, a circular Episomal SMN1 Transgene non-integrating is shown. A constitutive promoter drives the transcription of SMN1 mRN A, which exits the nucleus. In the cytoplasm, this mRNA is translated into green Functional SMN Protein clusters, leading to Maintenance of cellular homeostasis.

Panel C, titled Functional restoration and clinical impact, illustrates the physiological results. On the left, green protein clusters facilitate snRNP Assembly and RNA Splicing. An arrow points to a motor neuron axon terminal connecting to a muscle fiber, labeled Preserved NMJ Integrity. On the right, a large purple motor neuron is labeled Halted Motor Neuron Loss. A bracket at the bottom encompasses both outcomes, leading to the final text: Improved Clinical Motor Outcomes.

Navigate back to Figure 5..
